# Supplementary material for: Flexible and efficient handling of nanopore sequencing signal data with slow5tools
Source: Genome Biol. 2023 Apr 6;24:69. doi: 10.1186/s13059-023-02910-3 (PMC10080837; doi:10.1186/s13059-023-02910-3)
Supplement: Supplementary file 2 — Additional file 2. Review history. [file 13059_2023_2910_MOESM2_ESM.docx]

**1^st^ round**

**Reviewer 1**

The authors present slow5tools, a set of software tools for converting and manipulating nanopore signal data in the SLOW5/BLOW5 file format. The primary features are "f2s" and "s2f" which convert to and from FAST5s and SLOW5s/BLOW5s, along with "merge", "cat" "split", "index", "get", "view", and "stats". Several of these commands mirror those introduced by samtools, making it a familiar interface for bioinformaticians. The software seems well designed and efficient, and adherence to BASH-like design principles makes it easily extendable. The implementation details provided could also be a useful resource for developers of future file formats for other data types. However, I have a major concern with regard to novelty, and to a lesser extent I am doubtful of its long-term usefulness.

In terms of novelty, slow5tools was originally introduced in the SLOW5 paper (Gamaarachchi et al. 2022, Nature Biotechnology), where it supported conversion between SLOW5 and FAST5 files. While that paper didn't discuss slow5tools at length, it was included in the methods section and mentioned the "f2s", "s2f", "view", "merge", and "split" commands, and indexing was discussed elsewhere in the paper. There was also a mention of live FAST5 to SLOW5 conversion during sequencing, including an "extended data figure" on the topic. Given that conversion from a FAST5 file is the only way to obtain a SLOW5 file, it seems like these implementation details should have been included in the original SLOW5 paper. A few commands are discussed in this manuscript and not the original paper ("cat", "get", "stats", "quickcheck"), but these are not the main focus and I am not convinced they justify a separate paper.

My other concern is the lack of mention of the POD5 format, recently introduced by Oxford Nanopore as the successor to FAST5. There is no publication on the POD5 format and it was only supported by ONT's sequencing software as of January 2nd 2023, so this is not an egregious omission, but it is supported by ONT basecallers and an early version has been available since May 2022 (https://github.com/nanoporetech/pod5-file-format/releases/tag/0.0.1). ONT has announced their intention to transition entirely to the POD5 format, so conversion to and from this format by slow5tools is important for its long-term usefulness.

Minor comments:

"Normalized execution time" should be more clearly defined in the captions of Figure 2 and 3, presumably as execution time divided by the time for one process/thread. Figure 2 also contains the clause "relative to the number of parallel processes used by slow5tools", which I think refers to the x-axis but I initially thought referred to the relative units. Finally, it's inconsistently spelled "normalized" on the axis label but "normalised" in the caption.

Page 10 line 37: typo reads "BOW5", not "BLOW5"

**Reviewer 2**

The manuscript is a straightforward description of a software tool for ONT Fast5 files. The tool will likely be popular given the wide adoption of ONT and the awkwardness of the large Fast5 file generated by ONT sequencers.

In order to assess the software, we tried the tool on some of our Fast5 files. The slow5tools worked smoothly and fast. For 10 fast5 files (40000 sequences) using 8 processors it took 7.458 secs to convert the files to blow5 and 8.388 secs to convert back. Merging was also fast (3.040 sec). It compressed 942 Mb to 348 Mb, but reconversion led to a fast5 file size of 700 Mb. The authors may want to comment on why the file sizes may differ before and after conversion.

Overall, it seems like a good tool, although on first installation using conda it was not obvious that one needed to install a plugin. The error message was that "The fast5 file is compressed with VBZ but the required plugin is not loaded when trying to convert fast5 to slow5" and the error pointed me to the FAQ where the instruction for the installation could be found. It would be easier if this was specified in Github, quickstart right after the section starting with: "You can also use conda to install slow5tools as conda install slow5tools -c bioconda -c conda-forge."

There is also a minor error in figure 2. It states fast5-to-slow5, but the legend mentions blow5. This should be corrected.

**Authors’ response**

[[The authors present slow5tools, a set of software tools for converting and manipulating nanopore signal data in the SLOW5/BLOW5 file format. The primary features are "f2s" and "s2f" which convert to and from FAST5s and SLOW5s/BLOW5s, along with "merge", "cat" "split", "index", "get", "view", and "stats". Several of these commands mirror those introduced by samtools, making it a familiar interface for bioinformaticians. The software seems well designed and efficient, and adherence to BASH-like design principles makes it easily extendable. The implementation details provided could also be a useful resource for developers of future file formats for other data types. However, I have a major concern with regard to novelty, and to a lesser extent I am doubtful of its long-term usefulness.]]
We appreciate the reviewer’s thoughtful appraisal of our manuscript. Our responses to the specific issues they have raised are below.

1.1.
[[ In terms of novelty, slow5tools was originally introduced in the SLOW5 paper (Gamaarachchi et al. 2022, Nature Biotechnology), where it supported conversion between SLOW5 and FAST5 files. While that paper didn't discuss slow5tools at length, it was included in the methods section and mentioned the "f2s", "s2f", "view", "merge", and "split" commands, and indexing was discussed elsewhere in the paper. There was also a mention of live FAST5 to SLOW5 conversion during sequencing, including an "extended data figure" on the topic. Given that conversion from a FAST5 file is the only way to obtain a SLOW5 file, it seems like these implementation details should have been included in the original SLOW5 paper. A few commands are discussed in this manuscript and not the original paper ("cat", "get", "stats", "quickcheck"), but these are not the main focus and I am not convinced they justify a separate paper. ]]
The reviewer is correct that some of the core functions for data conversion and indexing were used in our original article introducing SLOW5 format1. While those were necessary to generate the SLOW5/BLOW5 files described therein, they were not the subject of the paper, which instead focused on the theoretical basis of SLOW5 format and demonstration of its performance advantages during data analysis. The current manuscript introduces slow5tools as a growing toolkit intended to facilitate adoption of SLOW5 format by nanopore users, and includes deep technical detail about the strategies used to ensure speed, efficiency, etc. Although it was in early development at the time of the original publication, slow5tools was not yet a mature toolkit and we were unable to describe slow5tools in detail due to the tight space limitations for a ‘Brief Communication’ paper in Nature Biotechnology.
There have been many updates to improve slow5tools core functionality and several new tools/options have been added since the original SLOW5 article was published.
This is captured in the change-log for the slow5tools Github, beyond v0.3.0. For example, since the submission of the current manuscript (June 2022), conversion between SLOW5 and ONT’s new POD5 format is now available (see 1.2 below) and a new tool named ‘skim’ has been added.
This is a key point: the novelty and impact of slow5tools is not limited to the functions described in the current paper; the toolkit will continue to grow, with new tools added as the field advances (similarly to samtools, bedtools, bcftools, etc). We are happy to admit that the current article does not describe a major new conceptual advance.
However, basic software toolkits like this have historically been among the most important, impactful and highly cited developments in the genomics field.
Actions: ‘skim’ added to Table S1; POD5 data conversion addressed (see 1.2 below).

1.2.
[[ My other concern is the lack of mention of the POD5 format, recently introduced by Oxford Nanopore as the successor to FAST5. There is no publication on the POD5 format and it was only supported by ONT's sequencing software as of January 2nd 2023, so this is not an egregious omission, but it is supported by ONT basecallers and an early version has been available since May 2022 (https://github.com/nanoporetech/pod5-file-format/releases/tag/0.0.1). ONT has announced their intention to transition entirely to the POD5 format, so conversion to and from this format by slow5tools is important for its long-term usefulness. ]]
The reason POD5 is not mentioned in our manuscript is because it did not exist – or existed only as a very preliminary prototype – at the time of our submission to Genome Biology (June 2022). ONT’s decision to develop POD5 was a direct response to our original SLOW5 publication. We have regularly consulted with the ONT developers working on POD5 and all agree that it will be important to enable conversion between POD5 and SLOW5, which will continue to exist as an open, community-centric format (whereas POD5 will be an internal format specifically designed to work on ONT hardware, for ONT in-house requirements).
Conversion between SLOW5 <-> POD5 format is now available and we have included this in our revised manuscript. However, because POD5 format is currently a prototype under active development, with major changes to the file structure occurring regularly, we have not yet integrated SLOW5 <-> POD5 conversion as a core utility within the main branch of slow5tools. Instead, we provide separate beta-version tools and usage instructions for POD5 conversion. At the point where POD5 is established as a mature, stable format, we intend to integrate these into slow5tools as core utilities (s2p and p2s). If interested, the reviewers can test POD5 conversion using our simple 's2p_conversion_alpha.sh' script, found here:
https://github.com/Psy-
Fer/project_blue_crab/blob/main/scripts/s2p_conversion_alpha.sh
Actions: Development of alpha-version tools for POD5 conversion; addition of a section describing POD5 conversion to the revised manuscript.

1.3.
[[ "Normalized execution time" should be more clearly defined in the captions of Figure 2 and 3, presumably as execution time divided by the time for one process/thread. Figure 2 also contains the clause "relative to the number of parallel processes used by slow5tools", which I think refers to the x-axis but I initially thought referred to the relative units. Finally, it's inconsistently spelled "normalized" on the axis label but "normalised" in the caption. ]]
We thank the reviewer for picking up these issues. All have been fixed and/or clarified in our revised manuscript.
Actions: Edits to legends for Figure 2 and 3 to improve clarity; corrected misspelling of ‘normalized’ throughout.

1.4.
[[ Page 10 line 37: typo reads "BOW5", not "BLOW5" ]]
This typo has been corrected.

Actions: Typo fixed on Page 10.

REVIEWER #2
[[ The manuscript is a straightforward description of a software tool for ONT Fast5 files. The tool will likely be popular given the wide adoption of ONT and the awkwardness of the large Fast5 file generated by ONT sequencers. ]]
We appreciate the reviewer’s thoughtful appraisal of our manuscript and we couldn’t agree more about the awkwardness of FAST5 files. Our responses to the specific issues they have raised are below.

2.1.
[[ In order to assess the software, we tried the tool on some of our Fast5 files. The slow5tools worked smoothly and fast. For 10 fast5 files (40000 sequences) using 8 processors it took 7.458 secs to convert the files to blow5 and 8.388 secs to convert back. Merging was also fast (3.040 sec). It compressed 942 Mb to 348 Mb, but reconversion led to a fast5 file size of 700 Mb. The authors may want to comment on why the file sizes may differ before and after conversion. ]]
We thank the reviewer for thoroughly testing the core utilities in slow5tools; it sounds like everything works smoothly.
The observation about FAST5 file sizes is a common one, and has been reported by us and others:
https://github.com/hasindu2008/slow5tools/issues/76
The explanation lies in the complex structure of the HDF5 format underpinning FAST5 files. Unlike a BLOW5 file, which will always be the same size, an HDF5/FAST5 file may differ considerably depending on how it was generated. We have found that ONT’s MinKNOW software (through which almost all FAST5 files are created) creates files with large quantities of ‘unallocated’ space, which typically inflates the file sizes by 10-20%. We don’t know why this happens, because MinKNOW is closed software, but it is highly reproducible. It is one of MinKNOW’s many mysteries. When converting from FAST5 -> BLOW5 -> FAST5, this ‘unallocated’ space is removed by slow5tools because it serves no purpose. No data is lost; the final file is simply packed more efficiently than the original.
Actions: we have added a FAQ to the slow5tools Github repository explaining this result (see FAQ#10).

2.2.
[[ Overall, it seems like a good tool, although on first installation using conda it was not obvious that one needed to install a plugin. The error message was that "The fast5 file is compressed with VBZ but the required plugin is not loaded when trying to convert fast5 to slow5" and the error pointed me to the FAQ where the instruction for the installation could be found. It would be easier if this was specified in Github, quickstart right after the section starting with: "You can also use conda to install slow5tools as conda install slow5tools -c bioconda -c conda-forge." ]]
We thank the reviewer for this suggestion and we have added this instruction to the ‘quickstart’ section on the slow5tools Github.
Actions: suggested conda installation instruction added to ‘quickstart’ on Github.

2.3.
[[ There is also a minor error in figure 2. It states fast5-to-slow5, but the legend mentions blow5. This should be corrected. ]]
This typo has been corrected.

Actions: Typo fixed in Figure 2 legend.

REFERENCES
1.Gamaarachchi, H. et al. Fast nanopore sequencing data analysis with SLOW5. Nat. Biotechnol. 40, 1026–1029 (2022).
